# Ictal Asystole in Drug-Resistant Focal Epilepsy: Two Decades of Experience from an Epilepsy Monitoring Unit

**DOI:** 10.3390/brainsci10070443

**Published:** 2020-07-12

**Authors:** Sara Casciato, Pier Paolo Quarato, Addolorata Mascia, Alfredo D’Aniello, Vincenzo Esposito, Roberta Morace, Luigi Pavone, Carlo Di Bonaventura, Mario Tombini, Giovanni Assenza, Giancarlo Di Gennaro

**Affiliations:** 1 IRCCS NEUROMED, 86077 Pozzilli (IS), Italy; sara_casciato@hotmail.com (S.C.); spleen333@libero.it (P.P.Q.); a_mascia@neuromed.it (A.M.); alfredod@vodafone.it (A.D.); vincenzo.esposito@uniroma1.it (V.E.); roberta.morace@yahoo.it (R.M.); bioingegneria@neuromed.it (L.P.); 2 Department of Human Neurosciences, Sapienza University, 00185 Rome, Italy; 3 Epilepsy Unit, Department of Human Neurosciences, Policlinico “Umberto I”, “Sapienza” University, 00185 Rome, Italy; c_dibonaventura@yahoo.it; 4 Unit of Neurology, Neurophysiology, Neurobiology, Department of Medicine, University Campus Bio-Medico, 00185 Rome, Italy; m.tombini@unicampus.it (M.T.); g.assenza@unicampus.it (G.A.)

**Keywords:** video-EEG monitoring, ictal asystole, temporal lobe epilepsy, cardiac pacemaker, syncope

## Abstract

Background: Ictal asystole (IA) is a rare event observed in people with epilepsy (PwE). Clinical and IA video-electroencephalographic findings may be helpful in screening for high-risk subjects. *Methods:* From all PwE undergoing video-EEG for presurgical evaluation between 2000 and 2019, we retrospectively selected those with at least one IA (R–R interval of ≥3 s during a seizure). *Results:* IA was detected in eight out of 1088 (0.73%) subjects (mean age: 30 years; mean epilepsy duration: 9.6 years). Four out of them had a history of atonic falls. No patients had cardiac risk factors or cardiovascular diseases. Seizure onset was temporal (*n* = 5), temporo-parietal (*n* = 1) or frontal (*n* = 2), left-sided and right-sided in five and two patients, respectively. In one case a bilateral temporal independent seizure onset was recorded. IA was recorded in 11 out of 18 seizures. Mean IA duration was 13 s while mean IA latency from seizure onset was 26.7 s. Symptoms related to IA were observed in all seizures. *Conclusion:* IA is a rare and self-limiting event often observed during video-EG in patients with a history of atonic loss of consciousness and/or tardive falls in the course of a typical seizure.

## 1. Introduction

Ictal asystole (IA), defined as an R-R interval longer than 3 s [1,2,3], is a rare complication occurring during a seizure, affecting about 0.3% of people with drug-resistant epilepsy who underwent video-EEG monitoring [1]. Ictal asystole is a potentially serious event, because it may cause syncope-related falls and injuries [2,3,4] that can be prevented by implanting a cardiac pacemaker [3,4,5]. Although IA may be clinically suspected when patients report a sudden loss of consciousness with falls, an established diagnosis requires simultaneous video-EEG and ECG monitoring.

Previously published case series and reviews of the literature suggest that people with temporal lobe epilepsy (TLE) may be at increased risk for ictal bradycardia and IA and, although a left-sided epileptogenesis has been suggested, larger series did not confirm a consistent lateralization [1,6].

Moreover, even if some electro-clinical features of IA have been described, details on the sequential electro-clinical changes observed during IA and their correlation with ECG are still lacking. A correlation was recently been demonstrated between atonia and duration of IA [7], but it is not clear whether IA duration is related to other symptoms of cerebral hypoperfusion and reperfusion.

With the aim to examine the prevalence of IA and its electro-clinical characteristics, we retrospectively reviewed a large sample of pre-surgical video-EEG long-term monitoring (LTM) evaluations of people with drug-resistant focal epilepsy.

## 2. Methods

### 2.1. Patients Selection

This study was conducted at the Epilepsy Surgery Center of the IRCCS NEUROMED (Pozzilli, IS, Italy). We reviewed a large cohort of consecutive subjects with drug-resistant focal epilepsy referred to our EMU for presurgical evaluation between January 2000 and 2019. We retrospectively selected all patients showing at least one IA during video-EEG prolonged monitoring.

IA was defined as an R–R interval of ≥3 s during an ictal epileptic event [4,9]. Patients with asystole during nonepileptic events (e.g., syncopal events) were excluded.

All subjects were evaluated with complete clinical assessment, neuroimaging, and continuous scalp video-EEG recording using the international 10–20 system for electrode placement, including supplementary anterior–inferior line temporal electrodes (SystemPlus Evolution, Micromed, Mogliano Veneto, Italy till 2011, then Nihon-Kohden Corporation, Nishiochiai, Japan) [10]. A single-channel recording was used to record the cardiac rhythm continuously. Anti-seizure medications (ASMs) were partially withdrawn during investigation according to clinical needs.

According to anatomo-electro-clinical principles, the seizure-onset zone was defined by multimodal information, including ictal clinical semiology, scalp EEG, and structural/functional brain imaging (MRI, FDG-PET).

All selected video-EEG were visually and qualitatively reviewed by two experienced epileptologists (G. DG. and S.C.), all data were discussed, and consensus was reached in all cases. Additional motor features associated with the asystole (e.g., sudden loss of postural tone [atonia], tonic and myoclonic movements) were assessed by video analysis.

The following aspects were reviewed and analyzed: (1) ictal clinical features; (2) topography and distribution of ictal EEG discharges at the beginning of a seizure and during IA as well as topography and distribution of post-ictal pattern; (3) temporal correlations of clinical symptoms with EEG and ECG changes; (4) durations and latencies of clinical and EEG features from both the seizure and IA onset; (5) seizures with and without IA in the same patient when data were available.

Additional cardiologic evaluation was performed in all subjects after the asystole was detected.

### 2.2. Statistical Analysis

A descriptive analysis was performed to calculate frequencies, means, and medians, as appropriate, of all variables of interest. The low number of patients who experienced IA prevented us from performing any further analysis.

## 3. Results

IA was detected in eight out of 1088 (0.73%) drug-resistant subjects who underwent video-EEG monitoring over a 19 year period of EMU clinical activity. Six patients were male, their mean age was 30 ± 7.4 years (range 18–38), mean age at seizure onset was 19.4 ± 10.3 years (range 8–35), and mean epilepsy duration was 9.6 ± 9 years (range 2–27). The median number of failed ASMs was two (range: 2–6) as well as the median number of current ASMs (range: 1–3). Four patients had a history of falls/drop attacks occurred after >5 years from onset of epilepsy. No patient had cardiac risk factors or history of cardiovascular diseases. Basal EKG did not show any rhythm disfunction.

Seizure onset was detected in temporal (*n* = 5), temporo-parietal (*n* = 1) or frontal lobe (*n* = 2), lateralized in the left and right hemispheres in 5 and 2 patients, respectively. In the remaining case, a bilateral temporal independent seizure onset was recorded. Interictal epileptiform discharges were localized in the same regions of epileptogenic zone (EZ) for each case.

Brain MRI disclosed focal lesions in six out of eight cases (three low grade tumors, two hippocampal sclerosis, one focal gliosis). The remaining two subjects had a focal epilepsy of unknown etiology.

Epilepsy surgery was performed in three cases, with favorable seizure outcome. Two patients refused surgery, while the remaining two cases were barred from surgery because the EZ was close to or overlapped eloquent areas. One subject was lost at follow-up. Pacemaker (PMK) implantation was offered and performed in all except one patient who underwent epilepsy surgery quickly.

By reviewing medical charts, ASMs regimens included sodium-channel blockers in 5 out of 8 subjects, but symptoms clearly related with possible IA (i.e., falls) were experienced already before sodium-channel blockers use. However, this type of ASM was discontinued in all cases.

Table 1 provides main demographic, electro-clinical, neuroradiological, and surgical data for each case.

**Figure 1:**
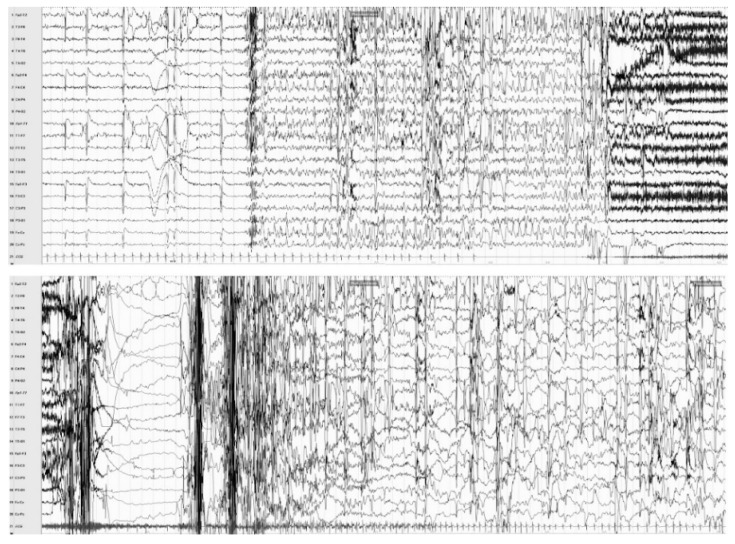
Subject #6, simultaneous video-EEG/EKG recording: ictal asystole during a focal seizure arising from the right frontal lobe. The EEG shows a rhythmical spike-and-wave discharge over right fronto-temporal regions and vertex, subsequently spreading to the homologous contralateral regions. The EKG documents a seizure-related change in cardiac activity, consisting of bradycardia, starting 8 s after seizure onset and evolving in asystole (duration: 20 s).

Among all selected video-EEG, a total of 18 seizures were recorded and reviewed. IA was observed in 11 (61%) seizures (see Figure 1 for an exemplificative case of a video-EEG documented IA). Mean IA duration was 13 s (±5.8; range 4–23). Mean latency from video-EEG seizure onset and IA was 26.7 s (±31.8; range 3–120).

In six out of eightt subjects, we had the opportunity to record focal seizures with and without IA: no clinical differences at the beginning of events were detected, while late motor patterns (characterized by myoclonic jerks, head/trunk drop) were evident when IA occurred.

As regard ictal clinical semiology 5/8 (62.5%) cases experienced aura, in all cases impaired awareness was detected. Autonomic signs (facial pallor/flushing/cyanosis, sweating, vomiting) were observed in 6/8 (75%) subjects. Only one event evolved from focal to bilateral tonic-clonic seizure.

Symptoms clearly related to IA were observed in all recorded seizures. The loss of tone appeared after a period of asystole usually lasting longer than 8 s and was associated with typical EEG changes seen otherwise with cerebral hypoperfusion (defined “burst suppression-like pattern” in the Table 2).

Table 2 describes in details ictal clinical features, EEG ictal pattern and IA data for each selected subject. 

## 4. Discussion

We reported a series of 8 subjects with IA disclosed from a retrospective review of large population of more than one thousand consecutive people with refractory focal epilepsy who underwent LTM for pre-surgical evaluation, along approximately 2 decades of clinical activity at our Center. The IA prevalence in our series was about 0.7%, substantially in line with literature reports and confirms that this event is rarely detected in an EMU. Indeed, although van der Lende et al. [1] in a systematic review showed that the mean prevalence of IA in all people with refractory focal epilepsy admitted for a LTM was 0.318%, Nguyen-Michel et al. [2] recently reported a higher prevalence (1.08%) in a large series of patients. Probably, publication bias, study design, and differences in selection criteria, as well as small sample sizes and variable definitions used for IA (R–R interval > 3 s, >4 s, no definition at all) could have led to an underestimation of the prevalence rate in the former review [1]. Two prospective studies with long-term implantable heart rhythm monitor devices (up to 2 years), reported a much higher IA prevalence, that was 5% and 21%, respectively [7,8]. By contrast, more recently, van der Lende et al. [11] in another prospective study, recruited people with refractory focal epilepsy without signs of IA and who had at least one focal seizure per month and implanted a loop recorder with 2 year follow-up. They did not identify any IA despite a high number of reported seizures. The most likely explanation is that the authors excluded those cases with a clinical suspicion of IA, suggesting that history taking is a powerful screening tool for asystole during seizures. Finally, the EEG-monitoring duration may play a role in evaluating the real IA incidence in a single subject, as IA could not occur during every seizure and may go unobserved during a relatively short-term EEG-monitoring. In this context, Hampel et al. [9] in a systematic review, showed that IA recurrence risk, calculated in 80 patients with 182 IA in 537 recorded seizures, amounted to 40%. Demographic and clinical subjects’ features such as age, sex, type and duration of epilepsy, side of epileptogenesis and duration of IA were not associated to short-term recurrence risk of IA. They concluded that, in case of clinically suspected IA, the recording of 1 or 2 seizures is not enough to rule out IA.

In line with the literature, in most of our patients with IA, seizure onset involved the temporal lobe (TLE or TLE plus). In fact, van der Lende et al. [1] reported that seizure onset zone, described in 78% of IA events, was temporal in 90% of them. As far the predominant temporal involvement is concerned, a possible explanation of its over-estimation is that, in many previously published series reporting video-EEG documented IA, as well as in ours, a selection bias could have played a crucial role since patients who underwent LTM for presurgical purpose are more often TLE, that is known to be the most common cause of drug-resistant focal epilepsy in adults [10].

Moreover, by reviewing our cases, the 75% of seizures showing IA were recorded from the left hemisphere. Left-side seizures have been previously reported to be more likely associated with the occurrence of IA or bradycardia [12,13,14,15]. Different cortical areas, such as the anterior portion of fronto-mesial cortex, the insular region and the nucleus ambiguous with the contribute of both mesial temporal areas have been proposed to have cardio-depressor properties [14,15,16,17]. Electrical stimulation studies of the left insula have been shown to produce cardiac depressor effects, leading to the hypothesis that cardiac parasympathetic function lateralizes to the left insular region, while the cardiac sympathetic function to the contralateral homologous region [14]. On the other hand, van der Lende et al. [1] did not confirm a consistent lateralization in the large group of ictal asystole and ictal bradycardia reported cases. Nevertheless, both localization and lateralization of IA remain nowadays still matter of controversy [18].

Moreover, Tényi and colleagues [18] in a systematic review of case report studies of patients diagnosed with IA, suggested that in new-onset IA, female gender and a preexisting heart condition could serve as predispositions in an otherwise benign epilepsy. Also, they speculated that in late-onset IA, male-predominant changes in neuronal networks in chronic, intractable epilepsy and an accompanying autonomic dysregulation serve as facilitating factors.

In most of our patients IA occurred in mid/end portion of the seizure, and the mean duration was >10 s. All IA events were self-limiting and did not require resuscitation maneuvers. Van der Lende et al. [1], after a literature analysis and review of 103 IA events, reported that mean duration of IA was 20 s, starting, on average, 30 s after seizure onset. All IA events but one resolved spontaneously. In the single near-SUDEP case, resuscitation was started after 44 s of cardiac arrest and was effective.

In this specific context, the question still unsolved is whether IA contribute to sudden death in epilepsy (SUDEP). Although a connection between ictal-bradycardia and SUDEP has often been suggested, available evidence suggest that such relationship is not probable [19]. Ryvlin et al. [20] evaluated data from 147 EMUs reporting findings in 16 SUDEP and nine near-SUDEP cases. Only two cases out of 20 evaluable episodes had focal seizures, while the remaining cases experienced generalized tonic-clonic seizures. Moreover, IA was observed only in one case during a focal seizure. In this study, deaths were associated with generalized tonic-clonic seizures and postictal cardiorespiratory arrest, thus considering bradyarrhythmia a secondary factor. A possible explanation is that IA, depending on its duration, may provoke diffuse cortical ischemia that may contribute to stop seizure, by reducing the efferent neural signals, primarily parasympathetic, deemed responsible sustaining IA [21].

So, in clinical practice, when should we suspect the possibility of IA in patients with known epilepsy? Several case reports [12,19], case series [3,4,5,6,7,8,9,10,11,12,13,14,15,16,17,18,19,20,21,22,23] and systematic reviews [1,18] suggest that change in ictal semiology with atonic loss of consciousness events and unexplained falls should alert clinicians about the possibility of IA occurrence. In fact, delayed loss of tone is distinctly uncommon in subjects with temporal lobe seizures but may inevitably occur in those cases with IA after a critical duration of cardiac arrest and cerebral hypoperfusion. Five out of 8 patients with IA in our series had a recent history of episodes with atonic loss of consciousness. High frequency of events may be best initially evaluated by video-EEG monitoring and low frequency of events by a 48 h Holter ECG, followed by a prolonged cardiac monitoring with implantable loop recorder [7,8,19], as needed, consulting cardiology team for evaluating seizure-unrelated cardiac arrhythmias. According to literature [3,24], the video-EEG analysis of IA episodes suggests a reproducible electro-clinical pattern characterized by loss of tone, often accompanied by diffuse myoclonic jerks and vegetative findings, such as facial pallor or flushing, occurring relatively late during the event, usually preceded by temporal lobe seizures’ habitual clinical features. Ictal EEG shows often a bilateral rhythmical discharge followed, several ss after IA onset, by generalized flattening, slowing or a “burst-suppression like” pattern, typically seen during cardiac arrest and cerebral hypoperfusion. This electro-clinical sequence may also help to differentiate it from the previously described temporal lobe syncope in which atonia and falls, probably due to the rapid spread of the ictal discharge with possible involvement of the pontine reticular formation, were seen at the onset of the event [3,25].

Although in routine clinical practice cardiac pacemaker implantation is considered a definitive treatment of IA, clear indications for this procedure are lacking, and careful analysis on the benefit/risk ratio of pacemaker implantation with patient and cardiology team should be considered [19]. In view of ictal asystole’s self-limiting course in most cases, a reasonable approach is to optimize treatment avoiding ASMs with pro-arrhythmogenic properties (i.e., sodium channel blockers) and to evaluate the opportunity of epilepsy surgery in selected cases. If these treatment strategies would not be applicable or not work, cardiac pacemaker implantation should be considered principally for reducing falls and injuries due to seizure-induced syncope [25].

## 5. Conclusions

Our study confirms that IA is a rare and often self- limiting event observed in people with focal epilepsy undergoing video-EEG monitoring. Clinical history review often discloses changes in ictal semiology with the occurrence of atonic loss of consciousness and falls. Lateralization and localization of epileptogenesis in subjects with IA is controversial but left temporal seizure onset has been more frequently reported. Video-EEG documented IA shows a typical electro-clinical pattern resembling that of cardioinhibitory syncope. Pacemaker implantation may reduce falls and injuries.

## Figures and Tables

**Table 1:** Demographic and general patient’s data.

Pts	Age (y)/Sex	Age at Seizure Onset (y)	Epilepsy Duration (y)	History of Drop Attack/Falls	IED (Side/Lobe)	MRI Findings	Baseline EKG	ASMs Failed (n°)	Current ASMs	Surgery (Outcome)	PMK Implantation
#1	18/M	16	2	no	Left/T	Left T Ganglioglioma (I WHO)	normal	2	LEV	Yes (seizure free)	No
#2	28/M	20	8	yes	Left/T	Left T Gangliocytoma (I WHO)	normal	4	CBZ, CLB	Yes (seizure free)	Yes
#3	28/F	8	20	yes	Bilateral (>left)/T	normal	normal	2	LEV, LTG	No	Yes
#4	38/M	34	4	no	Right/F	Right F DNT	normal	2	CBZ, TPM	No, refused	Yes
#5	37/M	35	2	yes	Left/T-P	Left T-P post- surgical gliosis	normal	2	VPA, TPM	No	Yes
#6	20/M	13	7	yes	Right/F	right HS	normal	2	VPA, LCS, ESL	No, refused	Yes
#7	36/F	9	27	no	Left/T	left HS	normal	5	CBZ, TPM, PB	Yes (rare seizures)	Yes
#8	37/M	20	7	no	Left/T-F	normal	normal	6	CBZ, LEV, PER	No	Yes

T = temporal; F = frontal; C = central; P = parietal; HS = hippocampal sclerosis; DNT = dysembriplastic neuroepitelial tumor; PNES = psychogenic non-epileptic seizures; PMK = pace-maker; IED = interictal epileptiform discharges; LEV = levetiracetam; PER = perampanel; VPA = valproate; CBZ = carbamazepine; LCS = lacosamide; ESL = eslicarbazepine; TPM = topiramate; PB = phenobarbital; n.a.: not applicable.

**Table 2:** Ictal electro-clinical data and asystole details.

	Ictal Clinical Features					EEG Ictal Pattern			Asystole Data		
Pts	Aura	“Nuclear” Semiology	Autonomic Signs	“Motor” Pattern	Post-Ictal Behavior	Seizure Onset	Evolution/Propagation	Post-Ictal	N° IA Events/Total V-EEG Recorded Seizures	Duration (Sec.)	EEG Onset →Asy (Sec.)
1	ascending epigastric nausea	behavior arrest; impaired awareness	vomiting pallor	diffuse tonic-clonic jerks	confusional state	Left T focal flattening/LVFA	T rhythmic theta activity	T theta-delta slowing	1/1	4	3
2	olfactive hallucinations	behavior arrest; impaired awareness; oral/gestural automatisms	flushing facial cyanosis	late diffuse myoclonic jerks	language deficit	Left T focal flattening/LVFA	T rhythmic theta activity	T theta-delta slowing	2/3	19/17	20/22
3	ascending fear/panic	behavioral arrest; impaired awareness	no	atonic falls	confusional state	Bilateral (>left) T rhythmic theta	diffuse rhythmic theta/PO activity	diffuse theta-delta slowing (burst suppression-like pattern)	1/1	23	20
4	no	behavior arrest; impaired awareness; grimace; right-side eyes deviation	facial cyanosis	head/trunk drop; diffuse myoclonic jerks asymmetric tonic posture	confusional state; post-ictal amnesia	Right F focal flattening/LVFA	rhythmic theta activity spreading over right F regions	diffuse theta-delta slowing	2/3	8/15	10/11
5	no	behavior arrest, impaired awareness; oral automatisms	no	bilateral tonic posture; falls	language deficit	Left T-P delta activity	diffuse rhythmic delta/PO activity	diffuse theta-delta slowing (burst suppression-like pattern)	1/2	14	33
6	epigastric pain	behavior/speech arrest; impaired awareness	vomiting pallor	trunk drop; late diffuse myo-jerks	confusional state; fear/panic reaction	Bilateral F (>right)	diffuse rhythmic delta/PO activity	diffuse theta-delta slowing (burst suppression-like pattern)	2/4	20/9	19/20
7	no	behavior/speech arrest; LOC; right arm dystonic posture;	flushing and up-eye-turn	trunk drop	no	Left T focal flattening/LVFA	rhythmic theta activity spreading over right F-T regions	background activity asymmetry (<in left hemisphere)	1/2	6	16
8	dizziness; ascending epigastric nausea	behavioral/speech arrest; impaired awareness	pallor sweating	late diffuse myo-jerks	confusional state	Left T-F rhythmic theta activity	rhythmic theta activity spreading over right F-T regions	diffuse theta-delta slowing (burst suppression-like pattern)	1/2	16	120

LVFA = low voltage fast activity; T = temporal; F = frontal; P = parietal; C = central; IA = ictal asystole.
